# Utilizing mobile digital radiography for detection of thoracolumbar vertebrae traits in live donkeys

**DOI:** 10.3389/fvets.2024.1322921

**Published:** 2024-02-29

**Authors:** Xinrui Wang, Muhammad Zahoor Khan, Ziwen Liu, Tianqi Wang, Xiaoyuan Shi, Wei Ren, Yandong Zhan, Changfa Wang

**Affiliations:** Liaocheng Research Institute of Donkey High-Efficiency Breeding, Liaocheng University, Liaocheng, China

**Keywords:** X-ray imaging system, equids, thoracolumbar vertebrae, body size, animal breeding

## Abstract

It has been well-established that the number of vertebrae is associated with body size and meat productivity. In current study we utilized a digital radiography (DR) technology to detect the number of thoracolumbar vertebrae in live donkeys. For this purpose, we introduced for the first time a groundbreaking device designed by our team for assessing thoracolumbar vertebrae number traits in equids, employing a sample of 1,000 donkeys sourced from five distinct donkey farms. This assessment incorporates a range of crucial body metrics, including body height, length, and various other measurements. Subsequently, our study determined the number of thoracolumbar vertebrae in 112 donkeys, utilizing the DR system. These findings were further validated through post-mortem evaluations conducted by slaughtering the donkeys. Our findings demonstrated a remarkable resemblance between the thoracolumbar vertebrae numbers visualized through the DR system in live donkeys and those obtained via slaughter verification. In conclusion, this research underscores the accuracy and effectiveness of the DR system for the detection of thoracolumbar vertebrae in live donkeys, which might be helpful for assessing the body size and meat productivity. We also recommended the utilization of DR system for counting thoracolumbar vertebrae in other animals in live state and could be a useful addition to livestock business industry for the prediction of body size and meat productivity efficiency.

## 1 Introduction

Donkeys play a pivotal role in the Chinese livestock industry, constituting a substantial part of the agricultural economy. However, since the turn of the millennium, China has witnessed a persistent decline in its donkey population, characterized by an annual decrease averaging 300,000 (1). This dwindling donkey population has resulted in a significant gap in the supply of donkey meat and hides, exacerbating the challenge of meeting the surging demand for donkey-related food products. Moreover, previous studies have revealed variations in the number of thoracolumbar vertebrae among domestic animals, including pigs, cattle, and sheep, and have linked these variations to production performance metrics such as body size, hide weight, and carcass weight (2–7). Unfortunately, most investigations into the number of thoracolumbar vertebrae have relied on post-mortem examination through slaughter (8–12), leading to the depletion of genetic resources. Consequently, the selective breeding of traits related to multi-thoracolumbar vertebrae has emerged as an economically significant aspect of the donkey industry, garnering increasing attention in breeding and local breed conservation efforts.

In studies pertaining to domestic animals, the correlation between the number of thoracolumbar vertebrae and associated traits has been explored. For instance, in European commercial pigs, the addition of an extra thoracic or lumbar vertebra was found to result in a 1.5 cm increase in body length (13). Similarly, research conducted by Zhang et al. on Mongolian sheep revealed that the addition of one more thoracic vertebra increased body length by 2.40 cm, while an additional lumbar vertebra led to a 3.50 cm increase, and both combined contributed to a substantial 5.90 cm enhancement in body length (5). In Kazakh sheep, those with an extra lumbar vertebra exhibited a 2.22 cm increase in body length and a 1.68 kg gain in carcass weight, whereas an extra thoracic vertebra resulted in a 2.93 cm increase in body length and a 1.90 kg increase in carcass weight (14). Similarly, in yaks, the presence of one more thoracic or lumbar vertebra led to an average spine length extension of approximately 7.6 cm, accompanied by an 8.29 cm^2^ increase in the area of oculomotor muscle (2). Additionally, the number of lumbar and thoracic vertebrae in donkeys was found to be significantly associated with body length, contributing to an average of 1.29 cm and 1.67 cm increase in body length for each additional lumbar and thoracic vertebra, respectively. These insights underscore the importance of these traits in donkey breeding, but the reliance on slaughter for data collection has hindered progress in livestock selection and breeding endeavors (8).

In the context of *in vivo* data acquisition technology, X-ray machines, commonly employed in human medical diagnosis, are increasingly finding application in the diagnosis of animal diseases. However, the existing fixed X-ray machines, often used in animal hospitals, present limitations when dealing with large animals, necessitating anesthesia during imaging (15). Furthermore, portable X-ray machines, while more versatile, often lack the power required for *in-vivo* detection of multi-vertebral traits in equids (16, 17). Notably, X-ray machines play a crucial role in diagnosing and treating skeletal disorders by precisely locating bone injuries, making them indispensable for life-saving procedures (18). However, their application in live breeding remains in its nascent stages.

To address these challenges, this study presents the special equipment for *in vivo* detection of multiple thoracolumbar vertebrae in live donkeys, centered on digital radiography (DR) technology. This groundbreaking device stands as the world’s first mobile X-ray machine explicitly designed by our team for the use of large animals, acknowledging the imperative need for X-ray imaging of substantial livestock. This innovation responds to the demand for *in-vivo* detection of multiple thoracolumbar vertebrae traits in donkeys, extending its utility to various breeding farms.

## 2 Materials and methods

### 2.1 Ethical statement

The experimental procedures regarding experiments and animals care were performed as per Animal Welfare and Ethics Committee of Institute of Animal Sciences, Liaocheng University under Ethical number (LC2019-1).

### 2.2 Animals and data collection

The study involved the acquisition of data from a total of 1,112 donkeys, comprising 1,000 donkeys sourced from five distinct donkey breeding farms and an additional 112 donkeys designated for verification during the slaughter process. In order to collect precise measurements, each donkey’s weight was meticulously assessed using a high-capacity digital electronic scale with a maximum capacity of 1,000 kg. Subsequently, a comprehensive set of body measurements was taken, including parameters such as body height, body length, thoracic circumference, thoracic depth, thoracic width, rump height, rump width, rump length, and cannon circumference. These measurements were systematically recorded employing measuring sticks and tape measures. Furthermore, crucial demographic information, such as age and sex, was meticulously documented for each donkey.

### 2.3 System construction

The integral apparatus designed for the *in vivo* detection of thoracolumbar vertebrae in donkeys is composed of four principal components:

- Digital Flat-Panel X-ray Imaging System: This component encompasses a digital flat-panel detector, featuring a single detection area measuring 43 × 43 cm^2^.
- Equids Retaining Device System (Radiography Bed): A versatile radiography bed, accommodating donkeys of diverse body sizes, forms an integral part of the system.
- Radiography Parameters for Equids: This component includes a set of radiography parameters specifically tailored to accommodate equids of varying body sizes and developmental stages.
- Polaris Stitching System: The Polaris system (QuantumTec Medical Devices Limited, Liaoning, China), developed by our group, specializes in stitching multi-thoracic and lumbar radiographic images of donkeys. It is noteworthy that we possess the intellectual property rights for the associated computer software.

The design of the radiography bed was informed by an exhaustive analysis of body measurements obtained from approximately 1,000 donkeys originating from five different donkey farms. This data was instrumental in determining the parameters governing the radiography bed’s design. Moreover, to account for the natural behavior and movement tendencies of donkeys, lateral holding devices were incorporated into the radiography bed to mitigate any undesired movement during the imaging process.

Radiographic parameters, adjustable according to the developmental stage and specific anatomical region of the donkey, are as follows:

Cervical and Thoracic Spine: 80–90 kV, 25–40mAs.
Lumbar Spine: 110–120 kV, 64–125mAs.
Donkey Foal or Colt: 60–80 kV, 20–40mAs.

The focal length employed is either 0.6 or 1.2, depending on the specific imaging requirements.

Key technologies within the image stitching system Polaris encompass image alignment and image fusion. Feature matching is achieved through the identification of common features across a given image set, subsequently allowing for the calculation of the transformation structure between images. This, in turn, facilitates the precise mapping of images by means of the calculated transformation structure. Intelligent algorithms are utilized to align feature points within superimposed images, and the selection of stitching seams is automated through the application of the graph cut method.

### 2.4 Verification of accuracy

To assess the accuracy of the system, a total of 112 healthy adult donkeys were randomly selected from the Liaocheng Dong’e National Black Donkey Breeding Base and Dezhou Yucheng Huimin Agricultural Technology Co. in the Shandong Province. The validation process was conducted as follows:

- Live donkeys were subjected to radiography using the DR system.
- Image stitching was performed using the proprietary Polaris software to ascertain the number of thoracolumbar vertebrae.
- Subsequently, the same donkeys underwent slaughter by means of electric stunning, allowing for the post-slaughter verification of the number of thoracolumbar vertebrae.
- A comparative analysis was carried out between the thoracolumbar vertebrae numbers obtained through *in vivo* radiography and post-slaughter examination to assess the accuracy of the DR system.

In order to ensure the robustness of the validation results, each of the aforementioned shooting operations was repeated three times, and any disparities observed in the number of thoracolumbar vertebrae between the *in vivo* and post-slaughter assessments were thoroughly investigated to identify root causes and prevent such discrepancies in future imaging processes.

### 2.5 Security protection

The primary operating environment for the custom-designed DR system employed in this study is situated outdoors. To ensure the safety of personnel and the environment, the system is equipped with a radiation dosimeter for real-time radiation monitoring. The system’s working range is carefully delineated, and a controlled area is established to limit the X-ray air absorption rate outside the boundary to no more than 20 μGyh-1. Prominent warning lines and warning signs are strategically placed for heightened safety awareness.

Operator safety is paramount, and as such, the operator’s protection workstation is fashioned from three lead plates. Additionally, operators are furnished with 0.35 mm thick lead protective clothing, offering effective shielding, capable of blocking 99.55% of X-rays emitted at 50 kV. These comprehensive safety measures collectively ensure the well-being of both operators and the surrounding environment during the operation of the DR system.

### 2.6 Operation specifications

**Figure 1:**
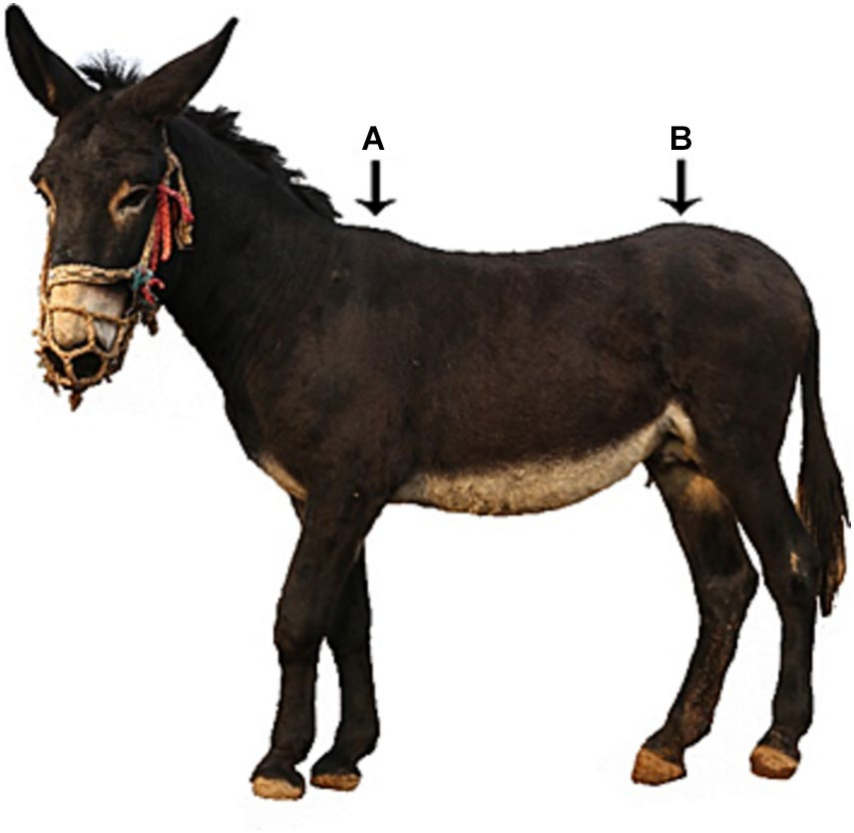
Schematic diagram of steel ball positioning technology. Position A is the first steel ball paste position, while position B is the last steel ball paste position.

After the donkey is secured, firstly, the donkey’s spine is manually partitioned, and the first steel bead is pasted at the withers (A) on the donkey’s spine, after which one steel bead is pasted at each 15 cm until it covers the sacral tuberosity (B) (Figure 1). Next, the operator wearing a lead suit adjusts the position of the beam limiter and flat detector. After the job is determined, it moves behind the lead plate to operate the laser emission control button for X-ray photography. After one image is taken, the above steps are repeated until the donkey’s spine is photographed. Finally, the shooting results were presented in the form of multiple images. Polaris software was used for image stitching to give a complete picture of the thoracolumbar spine. In addition, when adjusting, the electric lift type telescopic arm, slide rail layer, and slide table plate drives the beam limiter and flat detector to achieve up and down and back and forth movement, respectively.

### 2.7 Statistical analysis

The statistical analysis was performed using SPSS 26.0 (Statistical Product and Service Solutions, Version 26.0 Edition, IBM, Armonk, NY, United States) to obtain the design parameters of the photographic bed. Meanwhile, the number of thoracolumbar combinations was analyzed in 112 donkeys, and the percentage of each type was obtained. Measures of Central Tendency in Descriptive Statistics were used to analyze the body size data of donkeys to obtain the Mode, Median, and Mean, and thus determined the optimal design parameters for the radiography bed. Also, the number of thoracolumbar combinations of each type was obtained by Frequency Distribution, which in turn gave the percentage of each type of combination in this experimental population.

## 3 Results

### 3.1 Radiography bed

**Figure 2:**
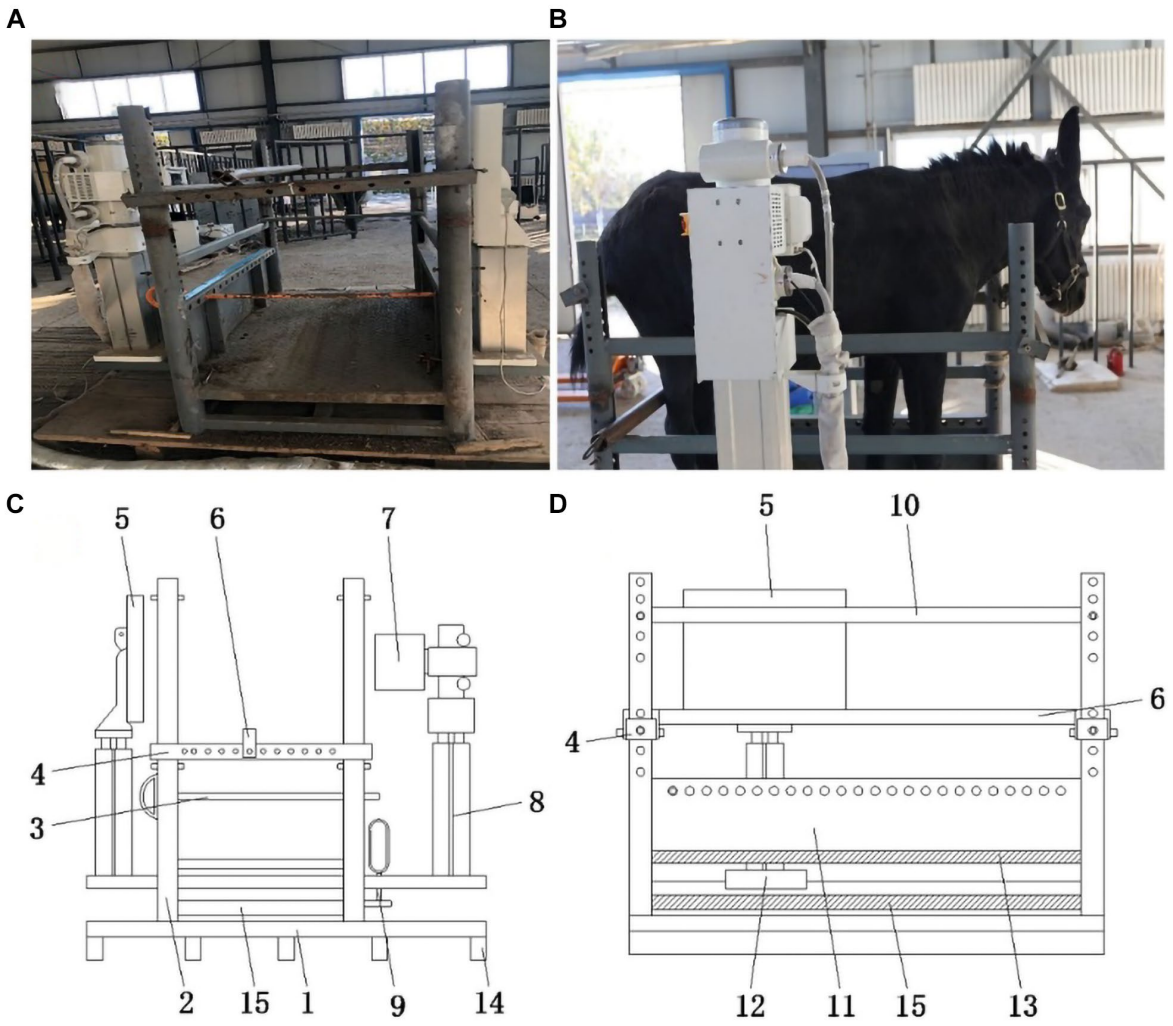
Radiography bed physical and model drawings. **(A)** Radiography bed front view. **(B)** Radiography bed physical side view. **(C)** Radiography bed model front view. 1-base plate; 2-supporting rod; 3-leg stop lever; 4-transverse retainer; 5-X-ray receiving flat panel; 6-longitudinal retainer; 7-X-ray tube; 8-lift type telescopic arm; 9-sliding rail lock; 14-longitudinal plate; 15-sliding rail layer. **(D)** Radiography bed model side view. 4-transverse retainer; 5-X-ray receiving flat panel; 6-longitudinal retainer; 10-stop lever; 11-retaining frame; 12-sliding table; 13-partition; 15-sliding rail layer.

Through the analysis of the donkey body size data (Table 1), we determined that the lifting range of the DR system lifting telescopic arm was 40 cm, the length of the radiography bed was 146 cm, the width was 88 cm, the height of the barrier was 150 cm, the size of the slide layer was 130 cm, and other customized data. Considering that the donkeys in intensive breeding are timid and resistant to stepping on the steps with a high drop (from the ground to the camera bed), a wooden ramp with the same height as the camera bed, 100 cm slope length, and 20° angle was set up at both ends of the camera bed. By analyzing the standing posture and movement of Dezhou donkeys, a hole for lateral barrier was installed in the middle of the barrier to prevent them from walking on the photography bed (Figure 2).

**Table 1:** Analysis table of body measurements for 1000 Dezhou donkeys.

Traits (cm)	Minimum value	Median	Maximum value	Mean value
Body height	85	133	158.5	131.74±0.34
Body length	61	131	164	129.04±0.47
Thoracic width	16	30	44	30.03±0.13
Thoracic depth	12.5	54.5	70	53.46±0.21
Rump height	40	136.5	163.5	134.06±0.44
Rump width	14	40	57.5	38.56±0.19

### 3.2 Radiographs

**Figure 3:**
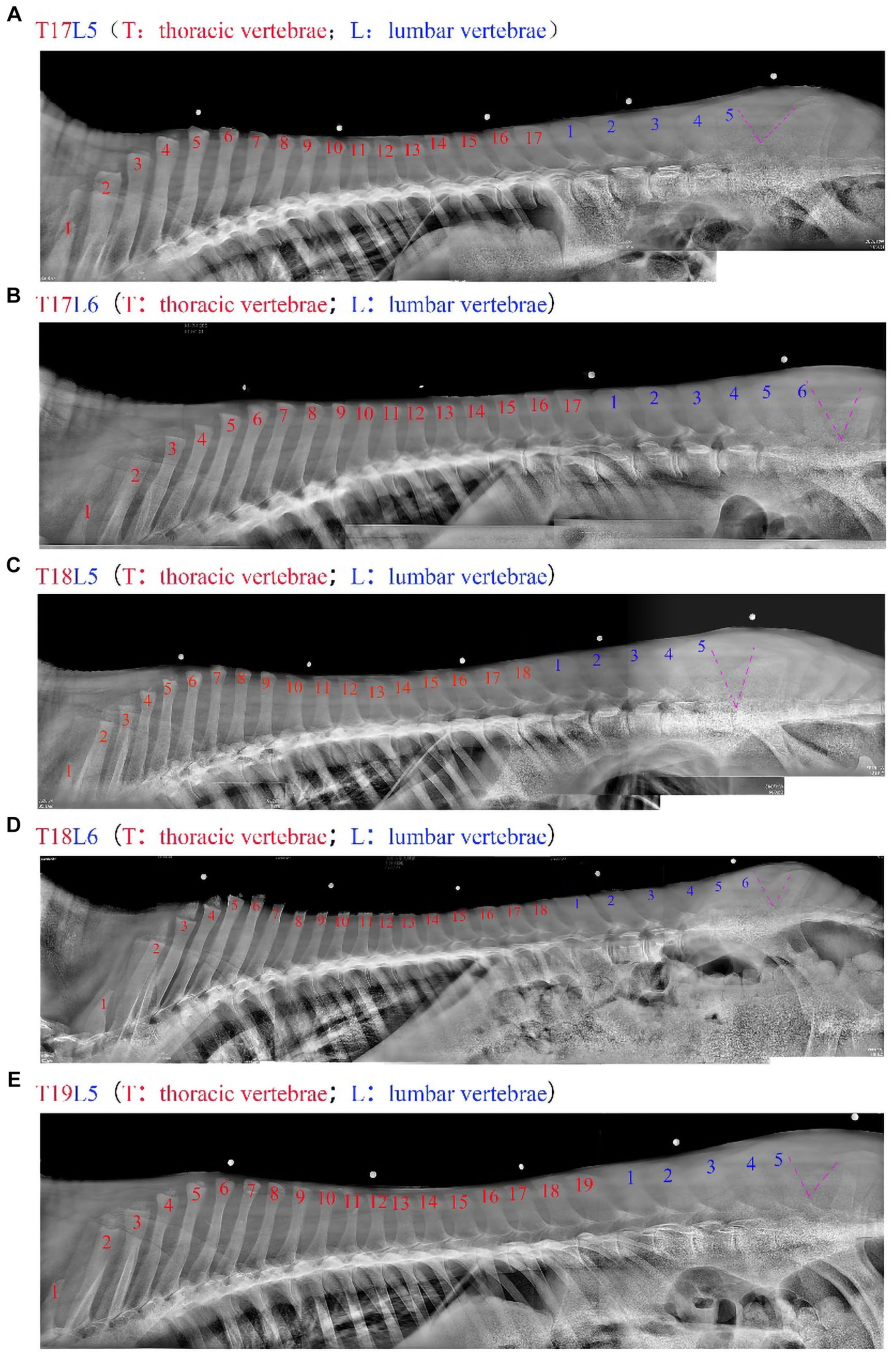
The thoracic and lumbar vertebrae of the donkey were spliced by the Polaris software, with the red part representing the number of thoracic vertebrae, the blue part the number of lumbar vertebrae, and the purple V shape representing the lumbar and caudal vertebrae dividing line. a-e represent 17 thoracic and 5 lumbar vertebrae (T17L5), 17 thoracic and 6 lumbar vertebrae (T17L6), 18 thoracic and 5 lumbar vertebrae (T18L5), 18 thoracic and 6 lumbar vertebrae (T18L6), and 19 thoracic and 5 lumbar vertebrae (T19L5), respectively.

After adjusting the shooting parameters to 85 kV and 25 mAs, a DR system was performed on the thoracolumbar spine of the donkey to obtain multiple radiographs. Polaris software is then used to align and fuse the images to get a complete thoracolumbar radiograph of the donkey (Figure 3).

### 3.3 Data comparison

**Figure 4:**
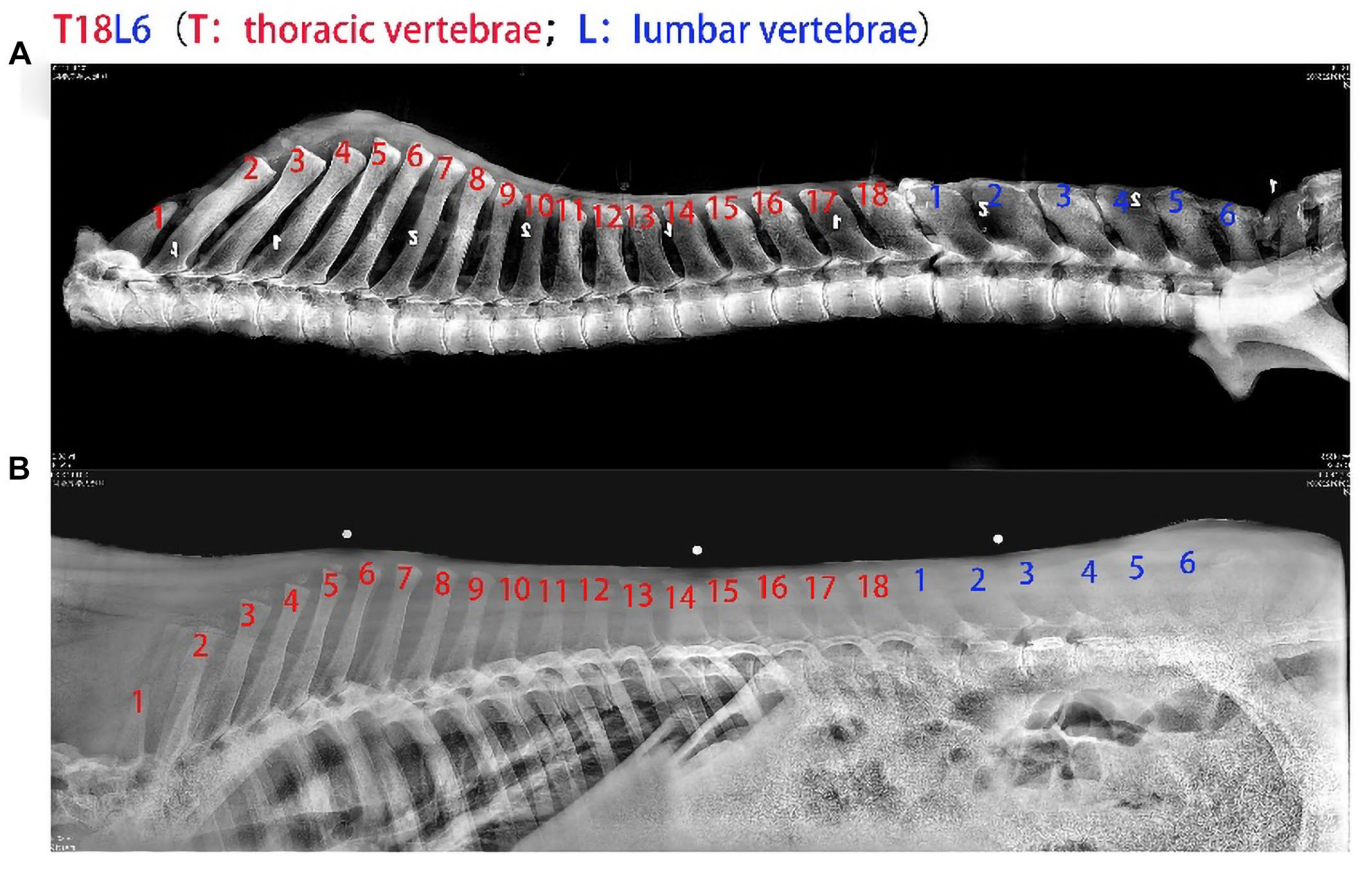
Comparison of the number of thoracolumbar vertebrae in live and slaughtered donkeys. **(A)** Graph of the number of thoracolumbar vertebrae of donkeys after slaughter. **(B)** Diagram of the number of thoracolumbar vertebrae in live donkeys.

The donkeys that underwent radiography were subjected to slaughter verification, and the slaughter-completed thoracolumbar vertebrae were radiographed again to ensure that the proof was accurately determined. Finally, the thoracolumbar vertebrae in live and post-slaughter radiographs were compared, and the results were consistent and 100% accurate after collating the data (Figure 4). Therefore, this equipment can meet the requirements of *in vivo* measurement of the number of thoracolumbar vertebrae, and the results are highly authentic.

### 3.4 Additional discoveries

When the number of thoracolumbar vertebrae in 112 donkeys was collated, it was found that there were five types of thoracic vertebrae combinations in donkeys (Table 2): the highest percentage of 18 thoracic vertebrae and 5 lumbar vertebrae (T18L5) types, up to 64.29%, and the lowest percentage of 19 thoracic vertebrae and 5 lumbar vertebrae (T19L5) classes, as low as 2.68%. The five thoracolumbar spine types (T17L5, T17L6, T18L5, T18L6 and T19L5) are consistent with the findings of Liu et al. (19).

**Table 2:** Combinations, quantities, and percentages of thoracolumbar vertebrae of 112 donkeys discussion.

Breed	Types	Quantities	Percentages
Dezhou Donkey	T17L5	5	4.46%
	T17L6	13	11.61%
	T18L5	72	64.29%
	T18L6	19	16.96%
	T19L5	3	2.68%

The five types are 17 thoracic and 5 lumbar vertebrae (T17L5), 17 thoracic and 6 lumbar vertebrae (T17L6), 18 thoracic and 5 lumbar vertebrae (T18L5), 18 thoracic and 6 lumbar vertebrae (T18L6), and 19 thoracic and 5 lumbar vertebrae (T19L5).

## 4 Discussion

The quantity of thoracolumbar vertebrae plays a pivotal role in influencing the growth characteristics of donkeys. These attributes, such as body weight and hide weight, are inherently linked to the animal’s physical size, which, in turn, correlates with the number of thoracolumbar vertebrae present in donkeys (19). Historically, the determination of thoracolumbar vertebrae count relied heavily on post-slaughter examinations, a method that was not conducive to facilitating *in vivo* selection and breeding processes (8–12). The present study has made a significant breakthrough in this regard by demonstrating that the DR system can accurately and consistently photograph the thoracolumbar vertebrae count of live donkeys. This novel capability aligns perfectly, with a 100% accuracy rate, with the data obtained through post-slaughter assessments. Consequently, the implementation of the DR system enables precise *in vivo* measurement of thoracolumbar vertebrae in live donkeys. This achievement addresses a longstanding challenge in the field, accelerating the progress of the donkey industry by facilitating enhanced breeding and selection strategies.

The DR system is used as the core of this equipment instead of computerized radiography (CR), primarily due to the following reasons. Firstly, the DR system has gained popularity in animal hospitals (20). Secondly, the DR system has matured in terms of data acquisition methods and data analysis systems. Thirdly, in terms of imaging capabilities, it offers advantages such as high-resolution imaging, image post-processing capabilities, imaging speed, and more (21, 22). Fourthly, DR systems require fewer X-ray measurements, significantly enhancing security. A study by Zhu, G. et al. in Hunan Province found that entrance surface dose (ESD) levels generally ranked as follows: X-ray machine > CR > DR (23). Lastly, operating the DR system is simpler and more effective.

Additionally, the traditional DR shooting system has been improved by transitioning from a vertical structure of the flat detector and beam limiter to a horizontal one (22). This modification allows for lateral X-ray work on donkeys while they remain upright, which is necessary due to the large size of donkeys and the difficulty of manually adjusting their body position. Moreover, since donkeys’ length exceeds the size of the flat detector, photographing all thoracolumbar vertebrae at once is impossible. Therefore, a segmented shooting method was employed, and a donkey multi-thoracolumbar radiograph stitching system (Polaris software) developed by our research team was utilized to integrate the numerous images. This system is eligible for computer software copyright. Furthermore, this equipment represents a movable DR system for *in vivo* detection of thoracolumbar vertebrae traits in equids, making it versatile in terms of time and space. Firstly, the flexible assembly of the radiography bed and conformation frame allows for accurate measurement of thoracolumbar vertebrae in short-lived equids of all ages. Secondly, the DR system can be easily relocated to different donkey breeding bases, supporting various donkey farms. Thirdly, donkeys subjected to live detection via the X-ray machine show no adverse reactions; the production performance of breeding males and females remains unaffected, and semen quality and embryo development are minimally impacted (24, 25).

To ensure the accuracy of the DR system’s shots, each individual was photographed three times. The number of thoracolumbar vertebrae obtained from our *in vivo* testing matched the data obtained from slaughter verification of 112 healthy adult donkeys, with 100% accuracy. Similarly, in fracture surgery, correct positioning of the fracture site with a C-arm X-ray system can reduce the risk of surgical failure and significantly improve surgical efficacy. Thus, the DR system ensures the accuracy of thoracolumbar data acquisition (26).

Based on this experiment and our group’s research, Dezhou donkeys typically have 17–19 thoracic vertebrae and 5–6 lumbar vertebrae (8, 19). Therefore, by using the DR system, we can effectively increase the proportion of donkeys with 24 thoracolumbar vertebrae in the offspring group, thereby boosting donkey meat and hide production, increasing farmers’ income, promoting the growth of the entire donkey industry chain, and facilitating industrial development. Moreover, our breeding work is carried out based on the existing thoracolumbar combination type of Dezhou donkeys, without artificially changing bone development or negatively impacting the growth of donkeys.

The adoption of this equipment significantly influences the financial benefits of the social market. According to our preliminary research, Dezhou donkeys with 24 thoracolumbar vertebrae produce approximately 12 kg more meat and 0.5 kg more fresh donkey hide compared to those with 22 thoracolumbar vertebrae (19). Considering the price of 110 Yuan/kg for fresh donkey meat and fresh donkey hide in the first quarter of 2022, the economic benefit per individual can increase by 1,375 Yuan. With an annual slaughtering capacity of 40,000 Shandong donkeys, this could result in an estimated revenue increase of 55 million Yuan. Through *in vivo* testing, we can identify excellent breeding males with 24 thoracolumbar vertebrae, collect semen to produce frozen semen, and distribute it nationwide to increase the number of thoracolumbar vertebrae in donkeys across the country. In a country where the annual donkey slaughter volume is 1 million head, this could potentially lead to an income increase of 1.375 billion Yuan. It is clear that the number of donkey thoracolumbar vertebrae can indeed generate substantial income for the donkey industry (27). The only limitation is that this equipment is custom-made for our group, making it costly and potentially unaffordable for an ordinary donkey breeding base. Addressing the imperative need to facilitate wider dissemination and accessibility of this equipment to the scientific community is a matter requiring immediate resolution.

## 5 Conclusion

In conclusion, the DR system represents a groundbreaking advancement as the world’s first device specifically designed by our team for the detection of multiple thoracolumbar vertebrae number traits in equids. Its capabilities set a new standard for the precise *in vivo* assessment of thoracolumbar vertebrae count in donkeys. By offering a rapid and accurate means of selection and breeding, it not only propels the growth of the donkey industry but also simplifies the challenges associated with breeding and the preservation of local donkey breeds. The DR system stands as a pivotal tool in advancing the field of equine research and husbandry, with far-reaching implications for the sustainable development and protection of donkey populations. Furthermore, the protocol and DR system employed in the present investigation may be applied for the assessment of thoracolumbar vertebrae in diverse live animal species, aimed at evaluating attributes such as body size and meat productivity traits, subject to minor adjustments *in camera* positioning materials.

## Data availability statement

The original contributions presented in the study are included in the article/supplementary material, further inquiries can be directed to the corresponding authors.

## Ethics statement

The animal study was approved by Animal Welfare and Ethics Committee of Institute of Animal Sciences, Liaocheng University. The study was conducted in accordance with the local legislation and institutional requirements.

## Author contributions

XW: Conceptualization, Data curation, Formal analysis, Investigation, Methodology, Resources, Software, Validation, Writing – original draft, Writing – review & editing. MZ: Data curation, Validation, Writing – review & editing. ZL: Investigation, Methodology, Writing – review & editing. TW: Data curation, Investigation, Methodology, Writing – review & editing. XS: Investigation, Software, Writing – review & editing. WR: Data curation, Investigation, Writing – review & editing. YZ: Conceptualization, Investigation, Methodology, Resources, Supervision, Visualization, Writing – original draft, Writing – review & editing. CW: Conceptualization, Funding acquisition, Investigation, Methodology, Project administration, Software, Supervision, Validation, Visualization, Writing – original draft, Writing – review & editing.
